# FGF21 Promotes Metabolic Homeostasis via White Adipose and Leptin in Mice

**DOI:** 10.1371/journal.pone.0040164

**Published:** 2012-07-06

**Authors:** Murielle M. Véniant, Clarence Hale, Joan Helmering, Michelle M. Chen, Shanaka Stanislaus, Jim Busby, Steven Vonderfecht, Jing Xu, David J. Lloyd

**Affiliations:** 1 Department of Metabolic Disorders, Amgen Inc., Thousand Oaks, California, United States of America; 2 Department of Pathology, Amgen Inc., Thousand Oaks, California, United States of America; Charité-Universitätsmedizin Berlin, Germany

## Abstract

Fibroblast growth factor 21 (FGF21) is a potent metabolic regulator, and pharmacological administration elicits glucose and lipid lowering responses in mammals. To delineate if adipose tissue is the predominant organ responsible for anti**-**diabetic effects of FGF21, we treated mice with reduced body fat (lipodystrophy mice with adipose specific expression of active sterol regulatory element binding protein 1c; Tg) with recombinant murine FGF21 (rmuFGF21). Unlike wildtype (WT) mice, Tg mice were refractory to the beneficial effects of rmuFGF21 on body weight, adipose mass, plasma insulin and glucose tolerance. To determine if adipose mass was critical for these effects, we transplanted WT white adipose tissue (WAT) into Tg mice and treated the mice with rmuFGF21. After transplantation, FGF21 responsiveness was completely restored in WAT transplanted Tg mice compared to sham Tg mice. Further, leptin treatment alone was sufficient to restore the anti-diabetic effects of rmuFGF21 in Tg mice. Molecular analyses of Tg mice revealed normal adipose expression of *Fgfr1*, *Klb* and an 8-fold over-expression of *Fgf21*. Impaired FGF21-induced signaling indicated that residual adipose tissue of Tg mice was resistant to FGF21, whilst normal FGF21 signaling was observed in Tg livers. Together these data suggest that adipose tissue is required for the triglyceride and glucose, but not the cholesterol lowering efficacy of FGF21, and that leptin and FGF21 exert additive anti-diabetic effects in Tg mice.

## Introduction

Fibroblast growth factor 21 (FGF21) regulates energy homeostasis in mammals [1], [2]. It is expressed predominantly in liver, white and brown adipose tissues (WAT & BAT) and pancreas [3]. In response to fasting or a ketogenic diet, FGF21 expression is induced primarily in the liver through the action of PPARα [4], [5]. Pharmacologically, FGF21 has become recognized as a modulator of glucose and lipid homeostasis *in vivo* [1], [6]. Recombinant FGF21 therapy corrected many metabolic perturbations in diseased rodent models and non-human primates [1], [6], [7]. In these different models, FGF21 reduced body weight, plasma and hepatic cholesterol and triglycerides levels, and increased oxygen consumption [1], [6]. Additionally, FGF21 improved the diabetic phenotypes of these animals by decreasing hyperglycemia, hyperinsulinemia, glucose intolerance, and increasing peripheral and hepatic insulin sensitivity [7]. Generation of FGF21 null mice confirmed the essential role of FGF21 in metabolic physiology [8], [9]. Mild obesity and adipocyte hypertrophy were observed in KO mice, indicating a crucial function for FGF21 in adipose tissue biology [8], [9].

FGF21 binds fibroblast growth factor receptor (FGFR) tyrosine kinases complexed with the co-receptor β-klotho [10], [11] and activates MAPK preferentially via FGFR1c/β-klotho [12]. It is reported that the restricted gene expression profile of β-klotho in liver, WAT and pancreas confers tissue specificity for the metabolic activity of FGF21 [13], [14]. Indeed, FGF21 rapidly induces FGFR and MAPK activation in liver and WAT tissues [15], suggesting that both tissues are FGF21 target organs. FGF21 was initially identified as a stimulator of glucose uptake in mouse 3T3-L1 adipocytes [1] suggesting a prominent role in adipose tissue, and participation in regulation of glucose metabolism in this tissue. Additionally, FGF21 is involved in the regulation of adipocyte lipolysis [5], [16], [17]. The liver may also participate in the glucose- and lipid-lowering effect of FGF21 since FGF21 suppresses hepatic glucose output and inhibits hepatic lipogenesis and triglyceride formation [6], [15]. Although, both liver and adipose tissue may participate in the pharmacological action of FGF21, the degree and nature of their contributions may differ. FGF21 has a 10-fold higher affinity to induce ERK-phosphorylation in adipose tissue than in liver, suggesting that FGF21 may preferentially target adipose tissue [15]. In addition, we observed that FGF21 acutely reduces blood glucose and insulin levels while it had no effect on cholesterol or triglyceride levels [15], thus FGF21 may induce separable responses on glucose and lipid homeostasis. The fact that FGF21 improves adiposity, lipid and glucose metabolism in a tissue-restricted manner and increases glucose uptake acutely in adipose tissues [15] prompted us to test whether the β-klotho expressing tissue, WAT, was necessary for FGF21 to elicit efficacy. [15].

Herein, we assess the pharmacological effects of recombinant murine FGF21 (rmuFGF21) in a mouse model with severely depleted adipose tissue levels. We generated lipodystrophy mice to replicate previously published aP2-nSREBP1c mice [18] and investigated the anti-diabetic and hypolipidemic efficacy of rmuFGF21. We found that apart from plasma cholesterol levels, rmuFGF21 was ineffective at improving glucose and lipid homeostasis in lipodystrophy mice. Further, adipose transplantation and leptin treatment restored FGF21 responsiveness in these mice.

## Methods

### Transgenesis

Lipodystrophy mice were generated at Amgen Inc. by cloning the mature human SREBP1c (residues 1–436) downstream of the mouse adipocyte fatty acid binding protein aP2 promoter. Briefly, a 1450 b.p. cDNA encompassing human SREBP1c was cloned into a transgenic vector containing the mouse 5.4 Kb aP2 promoter/enhancer genomic fragment and the SV40 small T intron and polyadenylation signal. The transgene was excised with *Sph2* and *Swa1* and purified for microinjection into FVB zygotes. Following confirmation of transgene overexpression by northern blot (Figure S1A), transgenic mice (Tg) were housed and bred at Charles River Laboratories (San Diego, CA), and maintained by continued backcrossing of Tg males to FVB females.

### Animal Procedures

#### Ethics Statement

All animal studies were approved by the Amgen Inc. Institutional Animal Care and Use Committee under protocol number 2006–00010.

Blood samples were collected from retro-orbital sinus of conscious mice, and used to determine blood glucose levels (OneTouch Basic glucometer or AlphaTRAK monitor as indicated). Plasma was collected in EDTA tubes. Body composition was determined using the EchoMRI apparatus. Glucose tolerance tests (GTT) were conducted in 12-hour fasted mice following IP injection of 2 g/kg glucose. Plasma cholesterol, triglycerides, and NEFA levels were measured using the Olympus AU400e Chemistry Analyzer (Olympus America, Inc; Center Valley, PA). Insulin and adiponectin values were determined by ELISA (Crystalchem and Millipore respectively). Plasma FGF21 levels were measured using an in-house ELISA [15]. Recombinant murine/human FGF21 and murine leptin were generated as previously described [15], [19].

### Animal Study Design I–effects of Chronic rmuFGF21

Plasma was collected from fed WT and Tg 10-week old male mice, based on glucose values, mice were separated into 3 groups (N = 8) per genotype such that the average intra-genotype blood glucose levels were similar. Each group was intraperitoneally (IP) injected with 1 or 10 mg/kg body weight/day rmuFGF21 or vehicle *bis in die* (BID) for 21 days at which point the mice were necropsied. Body composition was determined on treatments days 0, 7, and 14. A second fed plasma sample was collected after 14 days of treatment and GTTs were performed after 18 days of treatment (both 1 hour after dosing). At necropsy (day 21), liver, WAT (epididymal and inguinal) and BAT were excised, weighed and flash frozen for further analysis.

### Animal Study Design II–signaling Effects of rhuFGF21

WT and Tg mice 16–18 wks old were separated into vehicle and rhuFGF21 treatment groups (N = 5/group). Mice were administered a single IP injection of vehicle or 1 mg/kg body weight rhuFGF21 and were necropsied 15 minutes later. At necropsy, liver, and WAT (epididymal) were frozen in liquid nitrogen.

### Animal Study Design III–effects of Adipose Transplantation and rmuFGF21

Plasma was collected from a cohort of fed 14 WT and 28 Tg 15–16 week old male mice. Half of the Tg and all WT mice were sham operated, the remaining Tg groups underwent WT adipose transplantation. Donor WT animals were siblings to recipient Tg males, and sufficient WT epididymal adipose tissue was obtained from a single donor to increase Tg adipose mass by approximately 2 g (maximal amount for successful graft as determined in pilot studies). The adipose tissue was divided into 10 sections of approximately 200 mg each. Two small incisions were made along the ventral midline, and one incision was made on the dorsal side of each recipient. After implant placement, the incisions were closed with surgical glue. Fourteen days post surgery plasma was collected from all animals; the 14 WT mice were randomized into 2 groups such that the average blood glucose and adipose mass levels were similar. This randomization was also done for the Tg sham and Tg transplant groups of mice. Each group was treated with 10 mg/kg body weight/day rmuFGF21 or vehicle BID for 21 days at which point the mice were necropsied following decapitation. Body composition was determined before, immediately following, and 7, 14, 21 and 28 days after surgery. Fed plasma samples were collected pre-transplant, post-transplant pre-treatment and post-treatment (14^th^ day of treatment), and GTTs were performed on the 18^th^ day of treatment (both 1 hour after dosing). At necropsy, liver, WAT (epididymal, inguinal, and implants) and BAT were excised, weighed and fixed in 10% neutral buffered zinc-formalin, paraffin-embedded, H&E stained and examined by light microscopy.

### Animal Study Design IV–effects of rmuFGF21 and Rmuleptin

Plasma was collected from a cohort of fed Tg 11–16 week old male mice, based on blood glucose values, mice were grouped into 4 groups (N = 6) per genotype such that the average blood glucose levels were similar. Each group was treated with either 10 mg/kg body weight/day of rmuFGF21, muleptin, both or vehicle BID for 15 days. GTTs were performed on the 11^th^ day of treatment (1 hour after dosing). A second fed plasma sample was collected after 15 days of treatment prior to necropsy.

### Molecular Analyses

RNA was prepared from inguinal fat using the Qiagen RNeasy Mini Kit. Purified RNA was analyzed with the Qiagen RT-PCR Multiplexing system on an ABI Prism 7900. Cyclophilin A was used as the housekeeping gene (See Methods S1).

Tissue lysates were prepared in RIPA buffer (10 mM Tris-HCl pH 7.4, 100 mM NaCl, 1.0 mM EDTA, 1.0 mM EGTA, 10% Glycerol, 1% Triton X-100, 20 mM Na_4_P_2_O_7_, 1 mM PMSF, 2 mM NaVO_3_, 1 mM NaF, 0.1% SDS, 0.5% deoxycholate ) with protease (Complete EDTA-free, Roche), and phosphatase inhibitors (PhosSTOP, Roche). Samples were resolved on a 4–12% Bis-Tris gel and transferred onto nitrocellulose membranes. Proteins were detected using the following antibodies: anti-β-klotho (0.1 µg/mL, R&D systems, AF2619), anti-phospho ERK1/2 (1∶1000, Cell Signaling, 9101), anti-ERK1/2 (1∶1000, Cell Signaling, 9102), anti-phospho FGFR1 (1∶1000 Novus Biologicals, NB110-62076), anti-FGFR1 (Abcam, ab10646), anti-phospho FRS2-α (Cell Signaling, 3861), anti-FRS2 (1∶200, Santa Cruz Biotechnology, sc-8318), anti-phospho SHP2 (1∶1000, Cell Signaling, 3751), anti-SHP2 (1∶1000, Cell Signaling, 3752), and anti-β-actin (1∶5000, Sigma-Aldrich, A2228). The bands were visualized by chemiluminescence (Supersignal West Dura substrate, Pierce).

### Statistical Analysis

Data analysis was performed using GraphPad Prism 5 (GraphPad Software). A one-way ANOVA was employed to determine statistical significance and P-values were calculated using a Dunnett’s or Tukey posthoc test. A two-tailed student’s T-Test was used where indicated.

## Results

### Recombinant FGF21 Improves Glucose Homeostasis in Wildtype Mice but not in Lipodystrophy Mice

To evaluate the role of adipose tissue in eliciting anti-diabetic efficacy associated with FGF21 therapy, we generated mice with diminished adipose mass. These mice were intended to replicate those described by Brown and Goldstein [18], in which the mature N-terminal domain of the human SREBP1c (nSREBP1c) was overexpressed specifically in adipose tissues. Molecular characterization of the Tg mice (Figure S1A–B) demonstrated specific transgene expression in adipose tissues. Gene expression analysis of liver and adipose tissues (Figure S1C) showed multiple changes in the expression of metabolic genes. In WAT and BAT *Srebp1c, Ldlr* and *Hmgcr* were most upregulated by transgene overexpression, whereas in the liver *Pparg* was upregulated. In all these tissues *Fgf21* was upregulated between 10–25 fold. *Leptin* was the most downregulated gene. The phenotype of these Tg mice when bred on the FVB background was similar to that previously reported [18]. We observed similar body weights to WT mice, but dramatically decreased fat mass and commensurate increases in lean mass (Figure S2A–C), in addition we observed hyperglycemia (Figure S2D), and mild/sporadic glucose intolerance (Figure S2E–F). Additionally, Tg mice were severely insulin resistant (Figure S2G–H). Food intake was mildly increased in Tg mice compared to WT mice (Figure S2I). Tg mice exhibited hepatomegaly, and dramatically reduced WAT depot weights (Figure S2J). Triglycerides levels were lower, with little change in cholesterol levels (Figure S2K–L) in Tg mice. Taken together these mice present a similar model of lipodystrophy to that produced by Shimomura et al [18]. In contrast to the former model of lipodystrophy, we observed reduced triglycerides in Tg mice compared to WT mice. This could possibly be related to the difference in genetic background of the mice. Simomura *et al.* bred their mice on a B6/SJL background whereas here we breed to FVB mice. We found that WT FVB levels of plasma triglycerides are massively increased compared to WT mice of the original lipodystrophy mice (∼400 mg/dL vs. 74–123 mg/dL (Figure S1K and [18]). Futhermore we found that WT insulin levels are considerably higher than typical B6 values. It has been reported [20] that severely different phenotypes can be observed when the A-ZIP/F-1 transgene is bred onto B6 or FVB backgrounds, and these differences likely account for the differences we observe in our Tg mice.

**Figure 1:**
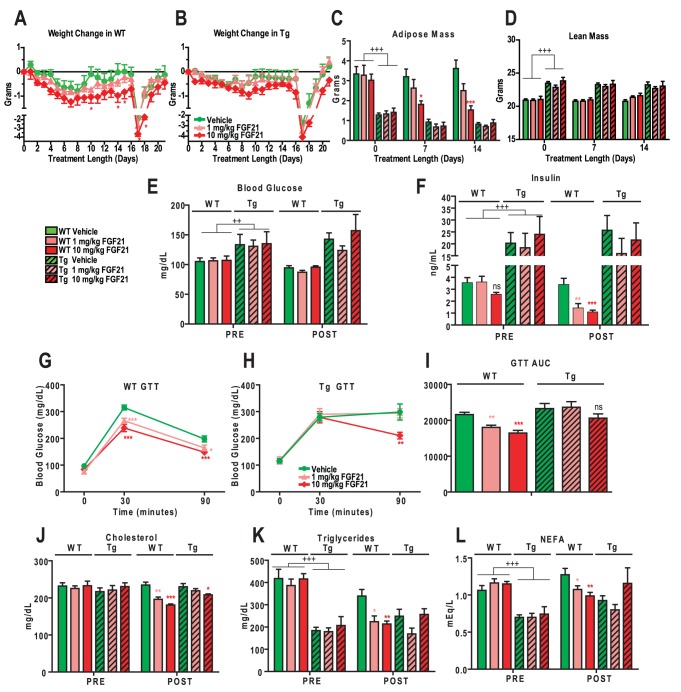
Lipodystrophy mice are resistant to anti-diabetic effects of recombinant FGF21 treatment. Recombinant muFGF21 (1 or 10 mg/kg, pink and red symbols respectively) or vehicle (green symbols) was injected BID in wildtype (WT - open bars) and aP2-nSrebp1c lipodystrophy (Tg - hatched bars) male mice as described in Animal Study Design I (Methodology). (A–B) Body weight was monitored daily. Dramatic drop in body weight on day 17 was caused by the 12 h fast for GTT. (C–D) Adipose and lean mass was recorded in WT and Tg mice at 0, 7 and 14 days of treatment. (E–F) Fed blood glucose (OneTouch Basic glucometer) and plasma insulin were measured in WT and Tg mice at the start (PRE) and the end (POST) of the treatment. (G–H) Glucose tolerance was measured on day 18 of treatment (OneTouch Basic glucometer). (I) Area under the curve (AUC) was calculated in WT and Tg mice. (J–L) Plasma lipids (cholesterol, triglycerides, NEFA – non-esterified fatty acids) were measured in WT and Tg mice at the start (PRE) and the end (POST) of the treatment. WT groups N = 8; Tg vehicle group N = 8; Tg FGF21 treatment group N = 7. * *P*<0.05, ** *P*<0.01, *** *P*<0.001 *vs.* vehicle-treated genotype-matched mice at same time point, ns – not statistically significant. ++ *P*<0.01, +++ *P*<0.001 *vs.* WT mice (T-Test).

The effects of 1 and 10 mg/kg of rmuFGF21 were assessed in WT and Tg mice. In WT mice, rmuFGF21 at 10 mg/kg reduced body weight (Figure 1A) but this effect was marginal in Tg mice (Figure 1B). Furthermore, rmuFGF21 reduced whole-body adipose mass (Figure 1C) and epididymal/inguinal fat pad mass (Figure S3A–B) in WT mice while no effects were observed in Tg mice. Lean mass, BAT, and liver weights were unaffected by rmuFGF21 in either WT or Tg mice (Figure 1D and Figure S3C–D). Blood glucose levels were unchanged in both WT and Tg mice following rmuFGF21 administration (Figure 1E), whereas rmuFGF21 reduced insulin levels (Figure 1F) and improved glucose tolerance in WT mice (Figures 1G and I). Despite the hyperinsulinemia in Tg mice, rmuFGF21 was ineffective in improving plasma insulin and glucose tolerance (Figures 1F, H–I). A minimal improvement in glucose tolerance was observed in Tg mice treated with 10 mg/kg rmuFGF21 (Figure 1H, 90 min.), but area under the curve (AUC) analysis did not reach statistical significance (Figure 1I).

Plasma lipids (cholesterol, triglycerides, and NEFAs) were all reduced by both doses of rmuFGF21 in WT mice (Figures 1J–L). Interestingly, only cholesterol levels were reduced by rmuFGF21 treatment in Tg mice, albeit to a lesser extent than in WT mice (Figure 1J).

### WAT Expression of FGF21 Receptor Complex in Lipodystrophy Mice

**Figure 2:**
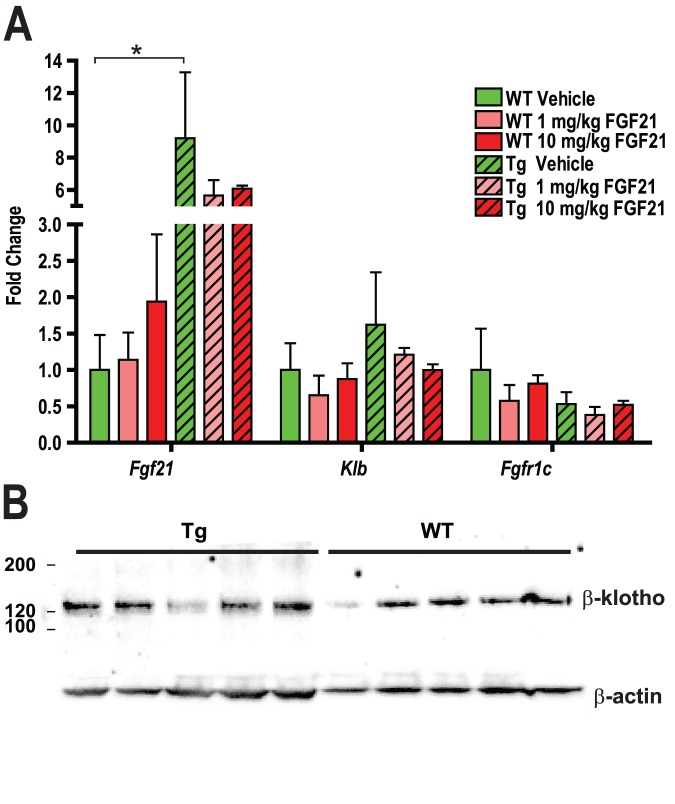
Increased *Fgf21* expression and normalβ-klotho protein levels in lipodystrophy mice. Recombinant muFGF21 (1 or 10 mg/kg, pink and red bars respectively) or vehicle (green) was injected BID in wildtype (WT - open bars) and aP2-nSrebp1c lipodystrophy (Tg – hatched bars) male mice for 21 days as described in Animal Study Design I (Methodology). (A) *Fgf21, Klb* or *Fgfr1c* expression levels were measured using semi-quantitative RT-PCR in RNA isolated from inguinal fat pads; N = 5/group. (B) Western analysis of β-klotho and β-actin was carried out on epididymal fat pad lysates from WT or Tg mice. * *P*<0.05 *vs.* vehicle-treated WT mice.

A defective FGF21 receptor complex in adipose tissue of Tg mice might lead to the lack of an improvement in parameters of glucose homeostasis following rmuFGF21 treatment. To investigate this possibility, we analyzed gene expression of *Fgf21*, *Klb* and *Fgfr1c* in the adipose tissue of WT and Tg mice (Figure 2A). Interestingly we found that *Fgf21* was overexpressed approximately 9-fold in Tg *vs.* WT adipose (consistent with Figure S1C). This was associated with a similar increase in circulating FGF21 levels 1.86±0.31 ng/mL *vs.* 0.31±0.04 ng/mL in Tg *vs.* WT, respectively (*P*<0.01). As receptor downregulation could account for an increase in *Fgf21* expression, the expression of the *Klb* and *Fgfr1c* components of the FGF21 receptor complex were also measured, but we failed to detect a difference in their expression (Figure 2A), however we had previously observed a small decrease in *Fgfr1c* in WAT (consistent with Figure S1C). To rule-out any post-translational downregulation of β-klotho, we performed immunoblot analyses and failed to find any differences in β-klotho protein levels of Tg *vs.* WT mice (Figure 2B). Treatment with rmuFGF21 did not significantly alter the expression of these genes.

### Defective FGF21 Signaling in Lipodystrophy WAT

**Figure 3:**
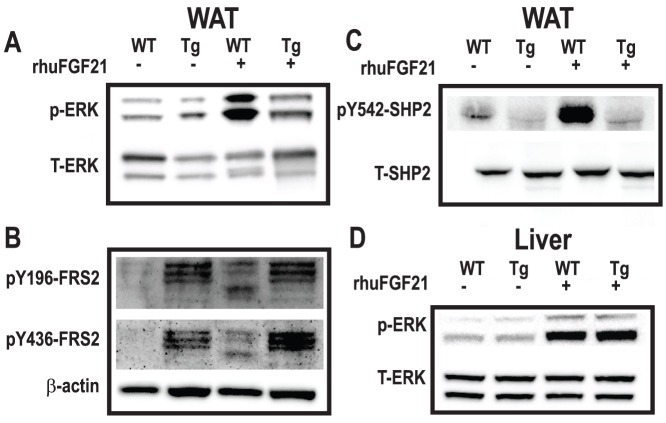
Impaired FGF21 signaling in adipose tissue from lipodystrophy mice. Wildtype (WT) and aP2-nSrebp1c lipodystrophy (Tg) male mice were treated with vehicle (−) or a single rhuFGF21 (+) injection at 1 mg/kg (Animal Study Design II – Methodology). White adipose tissue (WAT) samples were subjected to immunoblot analysis with antibodies directed against (A) ERK (extracellular signal-regulated kinase), (B) FRS2 (Fibroblast growth factor receptor substrate (C) SHP2 (Src homology domain 2 containing protein tyrosine phosphatase), 2), (D) ERK signaling was analyzed in liver samples. Antibodies directed against phosphorylated (p-) or total (T-) proteins of specific resides are noted alongside each panel.

To investigate the possibility that Tg adipose is somehow defective and resistant to FGF21, a single injection of 1 mg/kg recombinant human FGF21 (rhuFGF21) was administered in naïve WT and Tg mice, and FGF21-induced signaling was investigated in WAT and liver tissues (Figure 3). We chose rhuFGF21 as it is more potent than mouse FGF21, and anticipated to yield clear signaling responses [15]. Immunoblot analyses revealed expected ERK signaling in WT WAT with rhuFGF21, but a diminished phospho-ERK response in Tg WAT (Figure 3A). We further assessed the signaling of rhuFGF21 by analyzing Fibroblast growth factor receptor substrate 2 (FRS2) phosphorylation (Figure 3B). Injection of rhuFGF21 led to a clear induction of FRS2 phosphorylation, apparent as a larger 85 and smaller 75 kD band (due to multiple phosphorylation sites). In unstimulated Tg WAT, we observed FRS2 phosphorylation with a unique triple-banding pattern (approx. 80–85 kDa), which was unchanged in response to rhuFGF21, suggesting basal activation of the pathway, proximal to ERK activation. We next interrogated the FRS2 adaptor Src homology domain 2 containing protein tyrosine phosphatase (SHP2) (Figure 3C). In Tg WAT, SHP2 was minimally phosphorylated when unstimulated and remained unchanged in response to rhuFGF21. In liver, rhuFGF21 elicited similar ERK phosphorylation in both WT and Tg animals (Figure 3D).

### Adipose Transplantation in Lipodystrophy Mice Restores FGF21 Responsiveness

**Figure 4:**
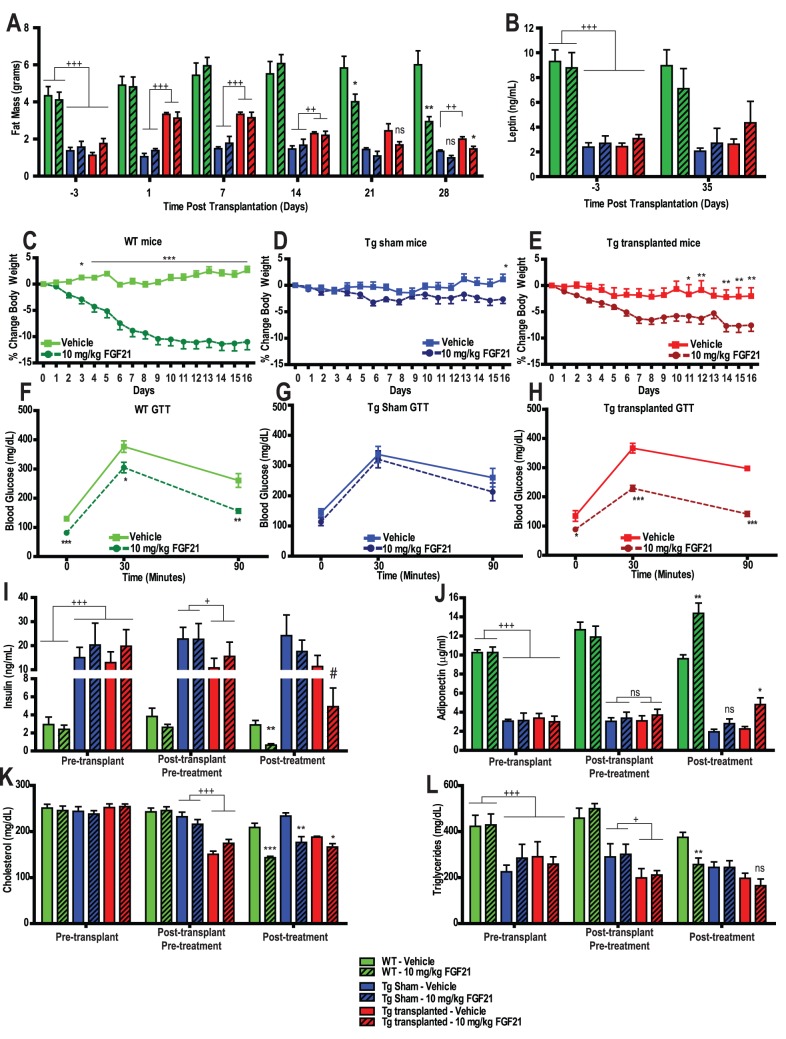
Adipose tissue transplantation restores anti-diabetic effects of recombinant FGF21 treatment in lipodystrophy mice. Approximately 2 g of wildtype (WT) adipose tissue was transplanted subcutaneously into aP2-nSrebp1c lipodystrophy (Tg) male mice (Animal Study Design III – Methods). Following a 2-week recovery period, WT sham (green bars/symbols), Tg sham (blue bars/symbols) and Tg transplanted (red bars/symbols) mice were treated with vehicle (open bars/solid line) or 10 mg/kg rmuFGF21 (hatched bars/dashed line) BID for 21 days. (A) Adipose mass was measured in WT and Tg mice before (−3 days), immediately after (1 day) and 7, 14, 21 and 28 days following adipose tissue transplantation. Adipose tissue mass was increased in the Tg transplanted mice after implantation and although declined by MRI analysis was still intact at necropsy. Fourteen days following adipose tissue transplantation, mice were treated with vehicle or rmuFGF21. (B) Plasma leptin was measured prior to, and at necropsy. (C–E) Percent change in body weight was calculated daily from the start of rmuFGF21 treatment. Days 17–21 were excluded due to dramatic weight changes following 12 hr fast for the GTT. (F–H) Glucose tolerance was measured after 18 days of rmuFGF21 treatment (AlphaTRAK glucose monitor). (I–J) Insulin and adiponectin levels were measured 3 days before adipose tissue transplantation (Pre-transplant), 14 days following sham or implantation surgery (Post-transplant Pre-treatment) and 32 days following surgery including 14 days of rmuFGF21 or vehicle injections (Post-treatment). (K–L) Plasma lipids (cholesterol and triglycerides) were measured in the same samples described in (I). N = 6–7 group. * *P*<0.05, ** *P*<0.01, ** *P*<0.001 *vs.* vehicle-treated genotype and surgery-matched mice at same time point;. + P<0.05, ++ P<0.01, +++ P<0.001 *vs.* comparator groups as indicated. # P<0.05. *vs.* the same mice Post-transplant Pre-treatment (all analysis by T-Test); ns – not statistically significant.

To determine if the FGF21’s anti-diabetic effects necessitate the presence of adipose tissue, we reconstituted adipose mass by implanting WAT from WT mice into Tg mice, and assessed the efficacy of rmuFGF21 in these animals. In pilot experiments (Figure S4) we determined that small (∼100 mg) pieces of WAT successfully grafted, and we could increase adipose mass by approximately 2 g (Figure S4A). We found that although the MRI apparatus detected the WAT following transplant, it was ineffective at monitoring the WAT transplants over time (>2–3 weeks, possibly caused by delipidation of WAT or additional cellular infiltrate) despite the fact that upon necropsy the fat pads were still intact and approximately the same size as their pre-implantation size. Nonetheless we considered the transplantation methodology successful due to the fact that leptin levels increased (Figure S4B), glucose and insulin levels had normalized (Figure S4C–D), and the steatosis in Tg mice had been reversed (Figure S4E *vs.* S4F). We observed that every transplanted fat pad was viable and had low grade inflammatory cell infiltrate (Figure S4G). Based on these data we investigated whether WAT transplantation could restore FGF21 efficacy. Immediately following implantation or sham surgery, the group average fat mass was increased from 1.4 g to 3.2 g in Tg transplanted (Tg–tx) mice (Figure 4A). At the completion of the study we measured leptin and found minimal increases in the Tg transplant mice in contrast to our pilot data (Figure S4B) and suspect that this may be related to differences in the 2 study designs; the pilot mice were implanted and allowed to fully heal, whereas the mice in this study were implanted, handled twice daily and tested for 2 weeks following transplantation. Nonetheless the transplantation methodology was successful in this study for several reasons, namely, plasma glucose, NEFAs, plasma FGF21 and liver weights were all lower in Tg–tx mice compared to Tg-sham mice (Figures S5A–D). Following the recovery period, WT sham, Tg sham and Tg–tx mice were treated with either vehicle or 10 mg/kg rmuFGF21. In mice treated with rmuFGF21, body weight was reduced in WT sham mice but not in Tg sham mice (Figure 4C–D), whereas body weight was significantly reduced in Tg–tx mice injected with rmuFGF21 compared to vehicle Tg–tx mice (Figure 4E). Glucose tolerance was improved in WT sham mice treated with muFGF21, but not in Tg sham mice also treated with rmuFGF21 when compared to vehicle-treated mice (Figures 4F–G). Importantly, Tg–tx mice treated with rmuFGF21 exhibited improved glucose tolerance *vs.* Tg–tx mice treated with vehicle (Figure 4H). These data strongly support a critical role of adipose tissue in eliciting the pharmacologic response of FGF21 on glucose homeostasis.

Consistent with the GTT data, similar effects with rmuFG21 were observed on insulin levels in the different groups of mice (Figure 4I). Recombinant muFGF21 reduced insulin levels in WT sham mice, but not Tg sham mice (Figure 4I; post-treatment), whereas it lowered insulin levels in Tg–tx mice (of note, this effect was only statistically significant when comparing the same mice to their pre-treatment insulin levels). Interestingly, we observed low plasma levels of adiponectin in Tg mice (Figure 4J), and transplantation did not alter these levels, however we observed an increase in rmuFGF21 treated WT mice and also Tg–tx mice only (no effect in Tg-sham mice) further supporting the beneficial effects of WAT transplantation and rmuFGF21 treatment.

Recombinant muFGF21 lowered circulating cholesterol levels in WT and Tg sham mice (Figure 4K). Recombinant muFGF21 exhibited a minimal reduction in cholesterol levels of Tg–tx mice (Figure 4K), possibly due to the extent of the direct effects of transplanting adipose tissue alone. Plasma triglycerides levels were also lowered in Tg–tx mice in response to adipose tissue transplantation alone (Figure 4L). In WT mice, rmuFGF21 lowered triglycerides levels as expected, whereas triglycerides were neither reduced in Tg sham nor Tg–tx mice administered rmuFGF21.

Liver weights were decreased by transplantation (Figure S5D) yet rmuFGF21 was effective in lowering liver weights only in Tg-sham mice. BAT weight was not affected by either transplantation or rmuFGF21 treatment in either WT or Tg mice (Figure S5E). Only WT adipose tissue weights were decreased in response to rmuFGF21, and transplantation in Tg mice had no effect on BAT and WAT (Figure S5E–F).

### Changes in *Peroxisome proliferator-activated receptor gamma* (*Pparg*) and *Fgf21* Expression in Adipose Transplanted Lipodystrophy Mice

**Figure 5:**
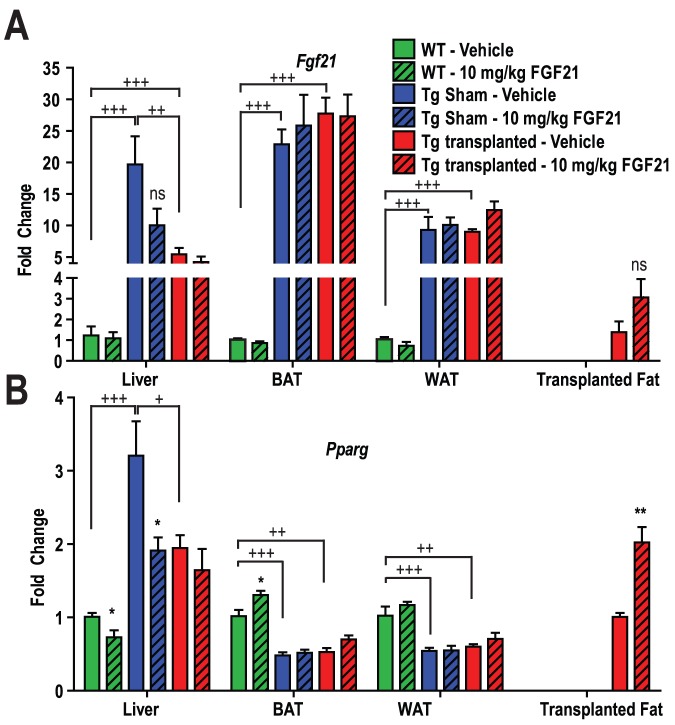
Changes in *Pparg* and *Fgf21* in adipose transplanted lipodystrophy mice. Relative quantitative RT-PCR was carried out on *Fgf21* and *Pparg* genes in tissues collected from animals presented in Figure 4 at terminal necropsy (Animal Study Design III – Methods). (A) *Fgf21* expression was increased in liver and white and brown adipose tissues (WAT and BAT) in Tg mice compared to WT mice. (B) *Pparg* expression was increased in the livers of Tg mice compared to WT mice and transplantation reduced liver *Pparg* expression in Tg mice in a similar manner to rmuFGF21 treatment, revealing a similar trend to *Fgf21* expression. *Pparg* expression was approximately 50% lower in Tg adipose tissues compared to WT mice. Treatment with rmuFGF21 increased the expression of Pparg in transplanted adipose only. N = 5 group. * *P*<0.05, ** *P*<0.01, *vs.* vehicle-treated genotype and surgery-matched mice at same time point (T-Test), ns – not statistically significant;. + *P*<0.05, ++ *P*<0.01, +++ *P*<0.001 *vs.* comparator groups as indicated (ANOVA).

Following transplantation and rmuFGF21 treatment in Tg and WT mice *Pparg* and *Fgf21* expression was measured (Figure 5 A–B). *Fgf21* expression was increased in liver and white and brown adipose tissues (WAT and BAT) 10–25 fold in Tg mice compared to WT mice. Transplantation reduced liver *Fgf21* expression in Tg mice, as did treatment with rmuFGF21 (Figure 5A). Treatment with rmuFGF21 had no effect on expression of endogenous *Fgf21* in adipose tissues. However it led to a slight increase in expression of the transplanted adipose. *Pparg* expression was increased in the livers of Tg mice compared to WT mice and transplantation reduced liver *Pparg* expression in Tg mice in a similar manner to rmuFGF21 treatment, revealing a similar trend to hepatic *Fgf21* expression (Figure 5B). *Pparg* expression in adipose tissues on the other hand were in opposition to those patterns observed for *Fgf21*; *Pparg* was approximately 50% lower in Tg adipose tissues compared to WT mice. Treatment with rmuFGF21 increased the expression of *Pparg* in transplanted adipose only.

### Histologic Findings in Adipose Transplanted Lipodystrophy Mice

Liver, WAT and BAT from WT and Tg mice which underwent transplantation or sham surgery and rmuFGF21 or vehicle treatment were analyzed histologically and morphologically (Figure S6A–B). The mice generated herein recapitulated the histological phenotypes previously published [18] albeit the livers presented milder hepatosteatosis. The BAT from Tg sham mice contained a heterogeneous population of adipocytes, some with a single, large vacuole and others with a more condensed, eosinophilic cytoplasm containing few, small vacuoles. Cells thought to be macrophages and/or fibroblasts were also present. The WAT from Tg sham mice was composed primarily of adipocytes that varied in size accompanied by scattered cells thought to be macrophages and/or fibroblasts. Livers from Tg sham mice showed swollen hepatocytes reflective of steatosis. Adipose tissue transplantation in Tg mice did not affect the morphology of BAT or WAT; however, the fat accumulation in liver was completely reversed (Figure S6A). Recombinant muFGF21 did not alter the histological phenotypes in WT sham (data not shown), Tg sham nor Tg–tx mice compared to vehicle controls. The lack of any observable difference in adipocyte size in WT or Tg–tx mice treated with rmuFGF21 is surprising given that the mice lose up to 10% in body weight, likely due to the fact the adipocytes are from lean (non-obese) mice. Nonetheless we continue to observe a reduction in total fat pad weight (Figure S5F–G).

A subset of adipose transplants from wildtype mice were excised to evaluate success of the tissue grafts (Figure S6B). Adipocytes were separated by cells that were thought to be macrophages and/or fibroblasts (Figure S6A). All or large portions of the explants appeared viable with the exception of one mouse in which the transplant was hemorrhagic and calcified. The data from this mouse were excluded from the study. Compared to our transplantation pilot study (Figure S4) we observed increased inflammation and more heterogeneity in adipocyte sizes in the explants from the current study. We attribute this to the difference in the 2 study designs; the pilot mice were transplanted and left to recover for 4 weeks, whereas the mice presented in Figure 4 were handled twice daily for injections and for regular measurements. This additional handling likely disturbed the grafting process of the transplanted WAT. Nonetheless we still had successful transplantation due to several results, namely, steatosis and liver weights were much improved (Figure S5D, S6), plasma glucose and NEFAs were improved (Figure S5A–B), as were insulin, cholesterol and triglycerides (Figure 4I, K–L). Thus we concluded transplantation was successful. Recombinant muFGF21 did not alter the appearance of the WAT explants.

### Leptin Restores FGF21 Responsiveness in Lipodystrophy Mice

**Figure 6:**
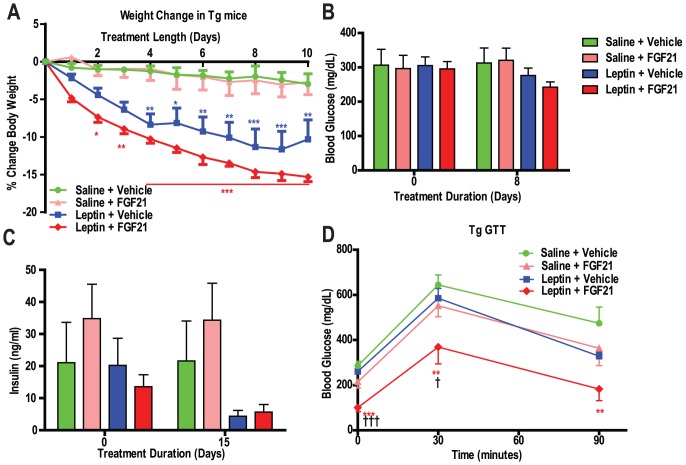
Leptin restores FGF21 resistance in lipodystrophy mice. Either rmuFGF21 (10 mg/kg, pink bars/symbols), muleptin (10 mg/kg/day, blue bars/symbols), both muFGF21+ muleptin (red bars/symbols), or vehicle (green bars/symbols) was IP injected BID in aP2-nSrebp1c lipodystrophy (Tg) male mice (Animal Study Design IV – Methodology). (A) Body weight was monitored daily, days 11–15 were omitted due to large changes at the time of GTT. (B) Blood glucose was measured prior to, and on the 8^th^ day of treatment (AlphaTRAK glucose monitor). (C) Plasma insulin was measured prior to, and on the 15^th^ day of treatment. (D) Glucose tolerance was measured on the 11^th^ day of treatment (AlphaTRAK glucose monitor). Groups of N = 6. * *P*<0.05, ** *P*<0.01, *** *P*<0.0001 *vs.* saline and vehicle-treated mice at same time point, † *P*<0.05, ††† *P*<0.001 *vs.* leptin and vehicle-treated mice at the same time point.

Since reconstitution of WT adipose tissue restored efficacy of rmuFGF21 (Figure 4H–J) we hypothesized that not only was the absolute amount of adipose tissue relevant but possibly a factor from adipose alone such as leptin could elicit some of the effects observed since Tg mice are severely hypoleptinemic and the Tx animals received WT adipose tissue (with intact leptin). We treated 4 groups of Tg mice with either rmuFGF21, recombinant murine leptin, both, or vehicle for 15 days. Recombinant muFGF21 was ineffective in reducing body weight, blood glucose or insulin levels, or in improving glucose tolerance (Figures 6A–D). Leptin significantly reduced body weight, and insulin levels, with a modest reduction in blood glucose and glucose tolerance. Impressively, rmuFGF21 and leptin co-administration resulted in significant reductions in body weight, blood glucose and insulin levels, and improved glucose tolerance. Furthermore, FGF21 and leptin in combination exhibited greater efficacy than leptin alone, and in the case of glucose tolerance it reached statistical significance (Figure 6D).

## Discussion

The selective expression of the FGF21 co-receptor β-klotho in adipose, liver, and pancreas indicates a clear association with tissues involved in control of glucose and lipid metabolism, but does not impart which tissue modulates the specific effects of FGF21 pharmacology. The data presented here suggests that WAT has a significant role in eliciting the anti-diabetic (glucose and insulin lowering) efficacy of FGF21, and exogenous leptin can potentiate FGF21’s actions.

We replicated a recognized model of mouse lipodystrophy [18] as a basis for our efficacy studies, primarily because it is a fully penetrant model of adipose deficiency, and has been used previously to assess the tissue-specific effect of anti-diabetic therapeutics [21]. Our initial observations of little-to-no effects of FGF21 in these mice were striking. A confounding aspect of the Tg mice is that, in addition to their reduction in WAT without changes in body weight, they replicate human lipodystrophy and exhibit multi-phenotypic consequences of adipose loss, namely, hepatomegaly/steatosis, insulin resistance, glucose intolerance, and chronic inflammation [22], and present hypotriglyceridemia which we attribute to the FVB genetic background Thus the lack of efficacy does not indicate directly that WAT is the pharmacological site of action of FGF21 since other tissues could also be resistant to FGF21 as a secondary consequence of reduced adipose mass; however, it does highlight the importance of adipose tissue *per se*. Another possibility is that these Tg mice have normal body weight and very little adipose tissue so they cannot become much leaner with FGF21 treatment. An important consideration in this study is that we cannot distinguish the anti-diabetic efficacy from weight loss caused by FGF21 since all improvements in glucose homeostasis were coincident with weight loss. In a previous study, we have shown that FGF21 can improve metabolic parameters independently of body weight changes [6].

Unexpectedly, the entire FGFR/β-klotho cell signaling cascade was altered in WAT from Tg mice. Proximally, β-klotho and FGFR1 mRNA were expressed normally in lipodystrophy WAT. Interestingly, pY-FRS2 (Figure 3B) and pY-SHP2 (Figure 3C) were increased and decreased respectively when compared to WT WAT. Furthermore this phosphorylation pattern in lipodystrophic WAT was unchanged in response to recombinant FGF21 (Figure 3B), collectively suggesting that FGFR1/FRS2 signaling was basally activated in WAT and cellular FGF21 resistance was observed in these mice. Adipose inflammation in this mouse model could account for the noted basal activation [22]. Indeed, we observed macrophage infiltration in lipodystrophic WAT (Figure S6) and thus the heterogeneous nature of this adipose could obscure the precise signaling events such that the observations are not reflective of adipocytes. Alternatively, if the changes observed in FGFR signaling are solely restricted to adipocytes, these data suggest increased FGFR1 in lipodystrophic adipocytes increases receptor phosphorylation and manifests in a unique pattern of FRS phosphorylation that impairs typical FGF21-induced signaling. The increase in plasma FGF21 is of interest and corresponds to the dramatic increase in *Fgf21* expression in liver, BAT and WAT. It has been reported that the liver accounts for most of circulating FGF21 [23], [24]. Despite the increase in *Fgf21* expression in these 3 tissues it appears that the circulating levels are mostly governed by the liver expression, since transplantation decreased the elevated FGF21 levels in Tg mice (Figure S5C) and correlates to a ≈75% reduction in *Fgf21* expression in liver only. The recent findings [23] that PPARγ mediates FGF21’s action promoted our investigation of *Pparg* expression in adipose tissues and liver in our mice. We observed that indeed Tg tissues exhibit differences in *Pparg* expression; levels of *Pparg* in WAT and BAT were reduced in Tg mice and this profile is consistent with the lack of efficacy of rmuFGF21, suggesting that PPARγ may play a role FGF21’s effects [23]. An important observation was that *Pparg* was increased only in transplanted WAT treated with FGF21– corresponding to the group of animals with restored efficacy.

We found separable features of efficacy following FGF21 therapy in the Tg mice, notably anti-diabetic *vs.* cholesterol lowering. Concomitant with a lack of improvement in insulin sensitivity, we continued to observe that FGF21 lowered plasma cholesterol levels (Figures 1J & 4K). Taken together with maintained FGF21 liver signaling in Tg mice (Figure 3D), it is possible that the liver is the target tissue for the cholesterol efficacy. Triglyceride levels were not coordinately lowered alongside cholesterol levels; in fact, Tg mice were resistant to FGF21-induced triglyceride lowering. It is possible that adipose tissue is required for triglyceride lowering efficacy. To support this, is the observation that FGF21 treatment in Tg-tx mice lead to a small (but non-significant) triglyceride reduction compared to vehicle-treated controls. Although this may indicate possible restoration of FGF21 responsiveness in non-WAT tissue, it highlights a dependence of WAT for FGF21 function.

Our finding that adipose tissue transplantation completely restored FGF21 responsiveness was of significant interest. The extent of the improvement in fat-transplanted Tg mice treated with FGF21 surpassed the typical effect observed with FGF21 administration in WT mice (Figure 4H. *vs.* 4F respectively), raising the possibility that the transplanted tissue not only restored WT amounts of adipose but also restored physiological functions itself. This perhaps is of most interest when comparing to a lack of restoration of TZD efficacy in WAT-transplanted A-ZIP/F-1 mice [25]. TZDs operate directly on WAT to improve insulin sensitivity and possibly implantation process leaves the tissue refractory to those direct actions (due to graft complications) whereas FGF21 co-operates with WAT to improve insulin sensitivity and glucose homeostasis, indeed we observed that adiponectin levels as a marker of insulin sensitity was only increased in WT and Tg–tx mice treated with FGF21. Additionally, the combination therapy of leptin and FGF21 improved all glucose and insulin measures (Figures 6A–D). The incremented effect in the GTT bore resemblance to that of the transplant + FGF21 group in the transplantation study (Figures 4H and 6D), suggesting that leptin, may be responsible for restoring part of the anti-diabetic efficacy of FGF21 in these mice. These data suggest WAT and leptin, but not liver dictate the anti-diabetic potential of FGF21. Consistent with this hypothesis is the finding that FGF21-induced signaling in the liver of lipodystrophic mice appears normal (Figure 3D).

Importantly, we observed a complete lack of FGF21-induced SHP2 phosphorylation in TG WAT. SHP2, when phosphorylated on Y542, couples FGF-induced FRS signaling to Grb2 binding and MAPK activation [26]. Despite our observation of increased FRS phosphorylation (Figure 3B), this did not correlate with increased SHP2 phosphorylation (Figure 3C). It is worth mentioning that FGF21 and leptin pathways possibly converge on SHP2 [27]. Since SHP2 is also activated in response to leptin-induced signaling, it will be worthwhile to investigate if leptin and FGF21 co-operate for typical MAPK activation.

FGF21 is efficacious in leptin null ob/ob mice and indicates that leptin is not required for FGF21’s anti-diabetic actions. In fact normal FGF21-induced signaling is observed in ob/ob adipose [24], [28], whereas we observe impaired FGF21 signaling in lipodystrophy WAT. Possibly, in Tg mice, not only does leptin exert additive effects to FGF21 on glucose and body weight lowering but also restores FGF21 resistance. It remains to be determined if leptin is responsible for the full restoration of FGF21 efficacy in Tg mice, or merely contributes to it; for example transplantation leads to mild increases in leptin levels, whereas leptin injections delivered supraphysiological doses. A worthwhile future experiment would involve the transplantation of ob/ob WAT into Tg mice to investigate whether leptin is required for the WAT rescue of efficacy.

In conclusion, our data show that lipodystrophic mice are resistant to FGF21’s effect on glucose homeostasis and adipose transplantation or supraphysiological leptin treatment can correct this impairment. It remains to be determined if leptin restores FGF21 anti-diabetic efficacy at the adipocyte, or whether other tissues/organs become FGF21-sensitive (such as the liver or BAT). Furthermore, the lipodystrophic mice provide a useful tool for elucidating the tissue-specific metabolic action of FGF21; the liver governs FGF21-induced cholesterol lowering, whereas the adipose tissue governs the FGF21-induced effects on triglyceride and glucose homeostasis. Together, these data have implications for the pharmacological additivity and interaction of FGF21 and leptin, and help to further understand the physiological mechanism of action of FGF21.

## Supporting Information

Figure S1 **Molecular characterization of the lipodystrophy model.** A) Northern analysis of transgene expression (probe to SV40 PolyA) in multiple tissues in wildtype (WT) and aP2-nSrebp1c lipodystrophy (Tg) mice (pooled RNA samples N = 3). Brown adipose tissue (BAT), Epididymal (Epi) and Inguinal (Ing) white adipose tissue (WAT) demonstrated very high levels of transgene expression. B) Relative qRT-PCR was carried out on WAT RNA samples to primers designed to amplify human nSrebp1c only. Snapshot of amplification plots demonstrates expression in the Tg WAT only. C) Relative qRT-PCR was carried out for WT and Tg RNA samples from WAT, BAT and liver against common metabolic genes, WT gene expression was expressed as a fold change of 1, N = 5 group. nd  =  not detected.
(EPS)

Figure S2 **Phenotypic characterization of the lipodystrophy model.** Wildtype (WT – green bars/symbols) and aP2-nSrebp1c lipodystrophy (Tg – red bars/symbols) male mice were monitored weekly for body weight (A), fat and lean mass by body composition MRI (B & C), and fed blood glucose using OneTouch Basic meter (D). At 13–14 weeks of age the mice were 12 h fasted and tested by a GTT using the OneTouch Basic meter (E). A second cohort of male mice were analyzed solely by GTT using the OneTouch Basic meter (F), illustrating the variability in glucose tolerance in the Tg mice *vs.* WT mice. A third cohort of male mice were analyzed by insulin tolerance test (ITT) at 10 weeks (G) and at 20 and 22 weeks for fed plasma insulin levels (H), revealing severe insulin resistance. Food intake was monitored in the same mice at 8–9 weeks (I). When sacrificed at 24 weeks white adipose tissues (WAT) and livers were weighed (J). Tg mice had lower plasma triglyceride levels (K) and normal to slightly lower cholesterol levels (L) compared to WT mice. N = 5–7 group, * *P*<0.05, ** *P*<0.01, ** *P*<0.001 *vs.* WT mice at same time point (T-Test).
(EPS)

Figure S3 **Tissue weight changes following FGF21 treatment in lipodystrophy mice.** Recombinant muFGF21 (1 or 10 mg/kg, pink and red bars respectively) or vehicle (green symbols) was injected BID in wildtype (WT - open bars) and aP2-nSrebp1c lipodystrophy (Tg - hatched bars) male mice as described in Animal Study Design I (Methodology). Following 21 days of treatment and testing as presented in Figure 1, the mice were necropsied. White and brown adipose tissues (WAT and BAT) and liver were harvested and weighed (A–D). WT groups N = 8; Tg vehicle group N = 8; Tg FGF21 treatment groups N = 7. * *P*<0.05, *** *P*<0.001 *vs.* vehicle-treated genotype-matched mice (ANOVA-Dunnett). +++ *P*<0.001 *vs.* WT mice (T-Test).
(EPS)

Figure S4 **Transplantation pilot in lipodystrophy mice.** Plasma was collected from a cohort of 7 fed wildtype (WT) and 13 aP2-nSrebp1c lipodystrophy (Tg) 13 week old male mice. Six Tg (blue bars) and all WT (green bars) mice were sham operated, the remaining 7 Tg (red bars) mice underwent WT adipose transplantation as described in Methods Section of Animal Study Design III. Fat mass was determined throughout the study (A). At 2 and 4 weeks post surgery plasma was collected from all animals and analyzed for leptin (B), insulin (C) and glucose levels using a OneTouch Basic meter (D). At necropsy liver and transplanted white adipose tissue were excised, fixed and H&E stained and examined by light microscopy. (E) Liver section of a Tg Sham mouse, (F) a Tg WAT transplant mouse, (G) morphology of the WT adipose transplant removed after 35 days of grafting from Tg mouse. All photographs were taken at a final magnification of 100×. * *P*<0.05, ** *P*<0.01, ** *P*<0.001 *vs.* comparator groups as indicated (T-Test or ANOVA as indicated).
(EPS)

Figure S5 **Additional physiological changes in lipodystrophy mice following transplant and FGF21 treatment.** Approximately 2 g of wildtype (WT) adipose tissue was transplanted subcutaneously into aP2-nSrebp1c lipodystrophy (Tg) male mice (Animal Study Design III – Methods). Following a 2-week recovery period, WT sham (green bars), Tg sham (blue bars) and Tg transplanted (red bars) mice were treated with vehicle (open bars) or 10 mg/kg rmuFGF21 (hatched bars) BID for 21 days. (A) Plasma glucose levels (using an AlphaTRAK glucose meter) and (B) non-esterified fatty acids (NEFA) were measured 2 days before adipose tissue transplantation (Pre-transplant), 14 days following sham or implantation surgery (Post-transplant Pre-treatment) and 32 days following surgery including 14 days of rmuFGF21 or vehicle injections (Post-treatment). (C–G) At necropsy (35 days post transplant) plasma was collected and FGF21 levels were measured and liver and brown and white adipose tissues (BAT and WAT) were weighed. N = 6–7 group. * *P*<0.05, ** *P*<0.01, *vs.* vehicle-treated genotype and surgery-matched mice at same time point. (T-Test). + *P*<0.05, ++ *P*<0.01, +++ *P*<0.001 *vs.* comparator groups as indicated (T-Test A–B, ANOVA C–G). ns – not statistically significant.
(EPS)

Figure S6 **Pathological features of lipodystrophic mice with and without adipose tissue transplantation.** A) Liver, epididymal white adipose tissue (WAT) and brown adipose tissue (BAT) were assessed in wildtype (WT) or aP2-nSrebp1c lipodystrophy (Tg) male mice 35 days following sham surgery or adipose tissue transplantation. The BAT and WAT from Tg sham mice contained a more heterogeneous population of adipocytes than seen in BAT and WAT from WT sham mice. This appearance was not affected by transplantation of WT fat into Tg mice. In contrast, the large vacuoles (fat) present in centrilobular to midzonal (c  =  central vein) hepatocytes of Tg sham mice were completely reversed by transplantation of WT fat into Tg mice. Livers from Tg mice receiving transplants were essentially identical to those from WT mice. An explant of WT WAT was excised from the subcutaneous area of a recipient mouse to reveal extent of adipocyte survival/immune infiltrate. Although adipocytes in the explants were heterogenous in size and structure, and in the amount of lipid contained within their single, large, cytoplasmic vacuole, the explants were largely to completely viable. Individual and clusters of adipocytes were separated by cells thought to be macrophages and/or fibroblasts. Tissues from Tg sham or Tg transplanted mice treated with rmuFGF21 were indistinguishable from the appropriate vehicle-treated controls. All photographs were taken at a final magnification of 200×; H&E. B) Photograph of a Tg mouse exposing the subcutaneous WAT transplants, to highlight the vascularization (arrow in inset).
(EPS)

Methods S1 Relative qRT-PCR from WT and Tg mice, Northern blot protocol of transgene insertion in multiple tissues, insulin tolerance test in WT and Tg mice, and food intake in WT and Tg mice.
(DOCX)
