# Supplementary material for: FGF21 Promotes Metabolic Homeostasis via White Adipose and Leptin in Mice
Source: PLoS One. 2012 Jul 6;7(7):e40164. doi: 10.1371/journal.pone.0040164 (PMC3391219; doi:10.1371/journal.pone.0040164)
Supplement: Methods S1 — Relative qRT-PCR from WT and Tg mice, Northern blot protocol of transgene insertion in multiple tissues, insulin tolerance test in WT and Tg mice, and food intake in WT and Tg mice. (DOCX) [file pone.0040164.s007.docx]

**SUPPLEMENTAL METHODS.**

**Relative qRT-PCR**

Relative FGF21 β-klotho (*Klb*), and *Fgfr1c* gene expression were analyzed using an ABI 7900 sequence detection system and using FAM^TM^ dye-labeled TaqMan® chemistry (Applied Biosystems, Foster City, CA, USA). All primers and TaqMan® probes (5’FAM- 3’TAMRA) used in the real-time quantitative RT-RCR was designed using the Primer Express sequence analysis software program (Applied Biosystems, Foster City, CA). Whenever possible, primers probe sets were designed to span exon-exon junctions to prevent detection of genomic DNA.

Each reaction was carried out with 100 ng of total RNA with 400 nM of primers and 200 nM probe in a 20 μl volume in 384 well plate format using the Quantitect Multiplex RT-PCR from Qiagen. Each RNA sample was ran in triplicate, in parallel with no-template and no reverse transcription controls. Cyclophilin A was used as the reference gene. Relative quantitation of gene expression was determined using the relative standard curve method.

Primer probe set sequences:

| **Gene** | **Forward Primer** | **Reverse Primer** | **Probe (5' 6-FAM 3' BHQ-1)** |
| --- | --- | --- | --- |
| *Srebp1a* | gac aca gcg gtt ttg aac ga | gcc agg gaa gtc act gtc ttg | tcg aag aca tgc ttc agc tta tca a |
| *Srebp1c* | gga gcc atg gat tgc aca tt | tca aat agg cca ggg aag tca | aag aca tgc ttc agc tta tca aca acc aag aca |
| *LDLr* | aaa gag gag gac act gtt cca aga | cgg gtg ttc ccc aat ctg t | cca gac cca gag cca tcg tag tgg |
| *Glut4* | cat ccc aca agg cac cct c | tca tgc cac cca cag aga ag | cta cgc tct ggg ctc tct ccg tgg |
| *FGF21* | gta cct cta cac aga tga cga cca a | tgc gcc tac cac tgt tcc a | aag ccc acc tgg aga tca ggg agg |
| *Pparγ* | gca gca ggt tgt ctt gga tgt | tca gtg gag acc gcc cag | ctt gct gaa cgt gaa gcc cat cga |
| *Fas* | cat gac ctc gtg atg aac gtg g | cgg gtg agg acg ttt aca aag | ccg tca ctt cca gtt aga gca gga caa gc |
| *Scd1* | cct tcg act act ctg cca gtg a | gcc atg cag tcg atg aag aa | acc gct ggc aca tca act tca cca c |
| *Acc1* | tga ggt cat cac cat cag cct | gtc cca gcc gga caa ggt a | tta cat gcc ggg cca ttg gta ttg g |
| *Acc2* | tgc atg gga tgc tga tca at | tgt gcc tgg aat cgc tta gc | cgc cct atg tca cca agg acc tgc t |
| *Hmgcr* | tgg gcc cca cat tca ctc t | gcc gaa gca gca cat gat ct | tga ttg gag ttg gca cca tgt c |
| *Klb* | cac cac ggc cat cta cat ga | cca ctc aaa gcc atc cag aag | cgc gtg ttt ggt tat acg gcc tgg a |
| *FGFR1c* | aat acc acc gac aag gaa atg g | agt tac ccg cca agc acg ta | tct acg gaa tgt ctc ctt tga gga tgc g |
| *Leptin* | cat ttc aca cac gca gtc gg | agc cca gga atg aag tcc aa | cca gtg acc ctc tgc ttg gcg g |
| *hSREBP transgene* | gtc tcc acc tcc tgc cac at | tgg agt ggg tgc agg ctg g | agc ggg ccg cag gca gcg ccc t |

**ADDITIONAL METHODS FOR SUPPLEMENTAL FIGURES**

**Northern Blot Protocol**

100-200 µg of Frozen Mouse Tissue was homogenized in 4 ml of Trizol (Life Technologies; Cat #15596-026) and purified according to instructions. Each of the representative tissue RNA samples were combined and further purified using a Qiagen Spin Kit (Qiagen; Cat #74104), including an on column DNAsing step with RNAse Free DNase Set (Qiagen; Cat #79254). 10 µg of total RNA was denatured and loaded on a 1.2% glyoxal/dimethylsulfoxide agarose gel and separated by electrophoresis (NorthernMax-Gly kit (Ambion / Life Technologies; Cat# AM 1946). The RNA was visualized by UV with EtBr staining to evaluate the quality of 28S and 18S ribosomal RNA bands. The RNA was transferred to a BrightStar-Plus Membrane (Ambion / Life Technologies; Cat# AM 10100) by downward capillary action using 20X SSC. The RNA was UV crosslinked to the membrane in a Stratagene UV Stratalinker 1800 set on Auto Crosslink.

A probe was made to the Tg SREBP-1c SV40 poly A 250 bp region by PCR. The probe was radiolabeled with Decaprime II Kit (Ambion / Life Technologies; Cat# AM 1455) using alpha-32P dCTP 6000Cu/mmol (Perkin Elmer; Cat# BLU013Z250UC) according to the instructions. 2x10E6 CPM / ml of probe and membrane were hybridized in UltraHyb (Ambion / Life Technologies; Cat# AM 8670) overnight in a roller bottle at 42C. Next day the membrane was rinsed (2X) in 2X SSC, 0.1%SDS at RT, and then washed (2X) in 0.1X SSC, 0.1% SDS at 42 ˚C, 15 min each. The membrane was wrapped in plastic and exposed to film in a cassette with intensifying screens at -80 ˚C.

**ITT**

Fed mice were injected IP at 1:00 PM with 1 U/kg of insulin (Humulin-R 100U) and blood glucose was measured at 30 and 60 minutes on an AlphaTRAK glucometer. A baseline glucose measurement was taken prior to the insulin IP injection.

**Food Intake**

Food was measured once daily for 3 consecutive days from single housed mice. The average intake over the 3 day period for each mouse was graphed.
